# Identifying Litchi (*Litchi chinensis* Sonn.) Cultivars and Their Genetic Relationships Using Single Nucleotide Polymorphism (SNP) Markers

**DOI:** 10.1371/journal.pone.0135390

**Published:** 2015-08-11

**Authors:** Wei Liu, Zhidan Xiao, Xiuli Bao, Xiaoyan Yang, Jing Fang, Xu Xiang

**Affiliations:** 1 Institute of Fruit Tree Research, Guangdong Academy of Agricultural Sciences, Guangzhou, China; 2 Key Laboratory of South Subtropical Fruit Biology and Genetic Resource Utilization, Ministry of Agriculture, Guangzhou, China; 3 Guangdong Provincial Key Laboratory of Tropical and Subtropical Fruit Tree Research, Guangzhou, China; 4 College of Life Sciences, South China Normal University, Guangzhou, China; 5 College of Horticulture and Forestry Science, Huazhong Agricultural University, Wuhan, China; USDA-ARS-SRRC, UNITED STATES

## Abstract

Litchi is an important fruit tree in tropical and subtropical areas of the world. However, there is widespread confusion regarding litchi cultivar nomenclature and detailed information of genetic relationships among litchi germplasm is unclear. In the present study, the potential of single nucleotide polymorphism (SNP) for the identification of 96 representative litchi accessions and their genetic relationships in China was evaluated using 155 SNPs that were evenly spaced across litchi genome. Ninety SNPs with minor allele frequencies above 0.05 and a good genotyping success rate were used for further analysis. A relatively high level of genetic variation was observed among litchi accessions, as quantified by the expected heterozygosity (He = 0.305). The SNP based multilocus matching identified two synonymous groups, ‘Heiye’ and ‘Wuye’, and ‘Chengtuo’ and ‘Baitangli 1’. A subset of 14 SNPs was sufficient to distinguish all the non-redundant litchi genotypes, and these SNPs were proven to be highly stable by repeated analyses of a selected group of cultivars. Unweighted pair-group method of arithmetic averages (UPGMA) cluster analysis divided the litchi accessions analyzed into four main groups, which corresponded to the traits of extremely early-maturing, early-maturing, middle-maturing, and late-maturing, indicating that the fruit maturation period should be considered as the primary criterion for litchi taxonomy. Two subpopulations were detected among litchi accessions by STRUCTURE analysis, and accessions with extremely early- and late-maturing traits showed membership coefficients above 0.99 for Cluster 1 and Cluster 2, respectively. Accessions with early- and middle-maturing traits were identified as admixture forms with varying levels of membership shared between the two clusters, indicating their hybrid origin during litchi domestication. The results of this study will benefit litchi germplasm conservation programs and facilitate maximum genetic gains in litchi breeding programs.

## Introduction

Litchi (*Litchi chinensis* Sonn.), a member of the Sapindaceae family, is an important fruit tree in tropical and subtropical areas of the world. The desirable characteristics of litchi fruit, such as bright color, exotic aroma, excellent taste, and rich nutritional value, make it very attractive and popular in international markets [1]. This fruit crop comprises a lucrative commodity and contributes significantly to the livelihood and economy of several million people in Southeast Asia.

Litchi is indigenous to southern China and has been introduced to other countries with tropical or subtropical climates over the past 400 years [2, 3]. Currently the leading litchi-producing countries in the world are China, India, Thailand, and Vietnam [4]. Specifically, China has the largest industry in the world, where litchi has been cultivated for more than 2,300 years [3, 5]. Due to the long history of litchi cultivation in China, rich litchi germplasm resources and abundant cultivars and strains have become established. At present, more than 500 accessions are preserved in the National Litchi Germplasm Gene Bank located at the Institute of Fruit Tree Research, Guangdong Academy of Agricultural Science, Guangzhou, China, which is the largest litchi germplasm gene bank in the world.

However, there is widespread confusion regarding litchi cultivar nomenclature. The same cultivars may have different names in different localities (synonyms), and different cultivars may have the same name (homonyms) [6]. Traditionally, litchi cultivar characterization in China has been based on morphological traits such as floral and fruit characteristics, and the harvest season [5]. The usefulness of this approach is limited due to the interaction of morphological traits with environmental conditions [7], and it is even more difficult to distinguish between closely related germplasm using only morphological characters. Therefore, litchi cultivar names must be standardized.

Furthermore, detailed genetic relationships among litchi germplasm in China are presently unclear. Precise evaluation of genetic diversity and population structures of germplasm collections contributes to information regarding (a) germplasm genetic variability, (b) parental combination identifications with maximum genetic variability for breeding programs, and (c) stratification and admixture avoidance when performing genome-wide association studies. Therefore, in order to facilitate optimum germplasm management and to establish appropriate breeding programs, a more comprehensive genetic characterization of litchi germplasm collections and their genetic relationships is urgently required.

Molecular genetic marker technology provides the most direct means for cultivar identification and genetic relationship analysis. A number of systems have been used in previous studies for litchi germplasm in China, including random amplified polymorphic DNA (RAPD) [8–11], amplified fragment length polymorphism (AFLP) [12, 13], sequence related amplified polymorphism (SRAP) [14], inter simple sequence repeat (ISSR) [15], and simple sequence repeat polymorphism (SSR) [16–18]. However, these studies were not based on broad genomic infrastructure and the marker numbers used were limited.

Recently, single nucleotide polymorphisms (SNPs) have become the most popular molecular genetic marker in plants. Compared to previous marker systems, SNPs have several advantages. Firstly, SNPs are the most abundant sequence variation class in plant genomes [19]. Secondly, SNP markers show a low rate of new mutations [20]. Thirdly, SNPs are mostly bi-allelic, and amenable to high-throughput genotyping and automation [21]. Fourthly, different SNP alleles are distinguished on the basis of the nucleotide present at a given position, avoiding any allele binning and allowing standardization and direct comparison of data arising from different laboratories. While SNP markers have been widely used in cultivar identification and genetic diversity investigations in many other crops, such as melon [22], grape [23], pummelo [24], olive [25], and pomegranate [26], the efficacy of using SNP markers for litchi genotype identification and diversity assessments remains to be investigated.

In 2010, the litchi genome sequencing and resequencing project was initiated by researchers from the China Litchi and Longan Industry Technology Research System (Project No. CARS-33) and many genomic resources have been developed for litchi including validated SNP sequences and high-density linkage maps (http://litchidb.genomics.cn/page/species/index.jsp). Deployment of available genomic resources and high-throughput tools will expedite cultivar identification standardization and the genetic relationship assessment of the litchi collection.

In the present study, we used a large set of 155 SNP markers in the molecular characterization of 96 representative litchi accessions from China. The aims were to (i) assess the genetic diversity of 96 litchi accessions collected from China; (ii) evaluate SNP use to differentiate litchi genotypes and to select a core set of SNP markers for litchi cultivar identification; (iii) survey the genetic relationships and population structure among those litchi accessions. The results will facilitate the establishment of a baseline for conservation and utilization of litchi germplasm in China.

## Material and Methods

### Plant material and DNA extraction

All the plant materials collected from National Litchi Germplasm Gene Bank and experimental stations/orchards were permitted by Institute of Fruit Tree Research, Guangdong Academy of Agricultural Science and China Litchi and Longan Industry Technology Research System, respectively. The plant materials used in this study did not involve protected species.

Ninety-six litchi accessions originating from five main litchi-producing provinces in China (Guangdong, Guangxi, Fujian, Hainan, and Yunnan) were used to represent the available genetic variation of litchi germplasm (Table 1). Young leaves of the 96 accessions were collected from the National Litchi Germplasm Gene Bank in Guangzhou and the experimental stations of the China Litchi and Longan Industry Technology Research System in Guangxi, Fujian, and Yunnan Provinces.

**Table 1:** List of 96 litchi accessions used in this study.

No	Cultivar	Origin	No	Cultivar	Origin
1	Sanyuehong	Guangdong	49	Caomeili	Guangxi
2	Dazao	Guangdong	50	Huangye	Guangxi
3	Zhimali	Guangdong	51	Qinzhouhongli	Guangxi
4	Xinxingxiangli	Guangdong	52	Jinzhong2	Guangxi
5	Zhuheli	Guangdong	53	Dadingxiang	Guangxi
6	Huaizhi	Guangdong	54	Longli	Guangxi
7	Chengtuo	Guangdong	55	Zhengfeng	Guangxi
8	Qiyueshu	Guangdong	56	Siliangguo	Guangxi
9	Ximili	Guangdong	57	Yuqilin	Guangxi
10	Shuangjianyuhebao	Guangdong	58	Huangrounuomici	Guangxi
11	Meiguilu	Guangdong	59	Siyuehong	Guangxi
12	Hongdenglong	Guangdong	60	Heli	Guangxi
13	Daniugu	Guangdong	61	Jizuili	Guangxi
14	Zhongshanzhuangyuanhong	Guangdong	62	Yuanhong	Fujian
15	Wusuzi	Guangdong	63	Bianli	Fujian
16	Nuomici	Guangdong	64	Lvhebao	Fujian
17	Budai	Guangdong	65	Xiangwan	Fujian
18	Hongli	Guangdong	66	Huangdijiu	Fujian
19	Jiefanghong	Guangdong	67	Lanzhu	Fujian
20	Shuidong	Guangdong	68	Xiapu	Fujian
21	Tongshachihuaizhi	Guangdong	69	Zhuangyuanhong	Fujian
22	Jinzhong	Guangdong	70	Kulin	Fujian
23	Maguili	Guangdong	71	Wuye	Fujian
24	Cuirou	Guangdong	72	Zhumuru	Fujian
25	Heiye	Guangdong	73	Xiafanzhi	Fujian
26	Baila	Guangdong	74	Magonghao	Fujian
27	Baitangying	Guangdong	75	Baibozaohong	Fujian
28	Jianjianghongnuo	Guangdong	76	Dachenzi	Fujian
29	Zengchengjinfeng	Guangdong	77	Xiaochenzi	Fujian
30	Xijiaozi	Guangdong	78	Wuyejiu	Fujian
31	Liuyuexue	Guangdong	79	Guilin	Fujian
32	Niangxie	Guangdong	80	Zhuzi	Fujian
33	Xianpoguo	Guangdong	81	Jidi	Fujian
34	Huidongsijili	Guangdong	82	Longyanben	Fujian
35	Zengchenggualv	Guangdong	83	Tongzi	Fujian
36	Mengtianshanzhi	Guangdong	84	Dongliuyihao	Fujian
37	Jiangjunli	Guangdong	85	Xinqiumili	Hainan
38	Chiye	Guangdong	86	Wuheli	Hainan
39	Yuhebao	Guangxi	87	Baipili	Hainan
40	Jianyeli	Guangxi	88	Huangpili	Hainan
41	Jianye	Guangxi	89	Edanli	Hainan
42	Goubei	Guangxi	90	Yutanmili	Hainan
43	Guangxitangbo	Guangxi	91	Anliangmili	Hainan
44	Tangbo	Guangxi	92	Nandaowuheli	Hainan
45	Baitangli 1	Guangxi	93	Yuanliyihao	Yunnan
46	Yanzhihong	Guangxi	94	Yuanlierhao	Yunnan
47	Lingshanxiangli	Guangxi	95	Hemaoli	Yunnan
48	Fuyu	Guangxi	96	Yuanyangerhao	Yunnan

The stability analysis of a selected 14 SNP set for genetic identification was conducted using 10 cultivars, representing a large variation in the cultivated litchi. Because litchi cultivars are clones, if the SNP markers used are stable, every plant of the same cultivar should show the same genotype for each SNP. Leaf material from 98 plants belonging to those cultivars was collected from National Litchi Germplasm Gene Bank in Guangzhou and 23 experimental orchards of the China Litchi and Longan Industry Technology Research System in Guangdong, Guangxi and Fujian Provinces (S1 Table).

Total DNA was extracted from fresh leaves of each accession using the modified CTAB method [27].

### SNP selection and genotyping

Among SNPs developed from litchi germplasm resequencing (unpublished work), three criteria were used for SNP selection: (1) evenly spaced across the seventeen litchi linkage groups; (2) minor allele frequency (MAF) above 5%; and (3) absence of other known SNPs in their vicinity. One hundred and fifty-five SNPs were selected based on the above criteria. The name, linkage group location, and alleles for the 155 SNPs are listed in S2 Table.

Genotyping assay for the selected SNPs was designed using the MassARRAY platform (Sequenom, San Diego, CA, USA). Loci that did not satisfy the Sequenom assay technical qualifications were discarded and the 148 loci that met all requirements were genotyped on the MassARRAY platform (Sequenom, San Diego, CA, USA) according to the manufacturer’s instructions, at BGI (Shenzhen, China).

### Data analysis

#### Polymorphism and discrimination power of SNP markers

Key descriptive statistics for measuring the informativeness of SNP markers were calculated, including minor allele frequency (MAF), observed heterozygosity (Ho), expected heterozygosity (He), and polymorphism information content (PIC). All of the estimates were calculated using PowerMarker v.3.25 software [28].

For duplicate identification, pair-wise multi-locus matching was applied among individual accessions using GenAlEx 6.5 [29]. Accessions with different names but that were fully matched at the genotyped SNP loci were declared as synonyms, somatic variants (sports), or misnomers. GenAlEx 6.5 was also used to show the relationship between increased genotype numbers and the addition of SNP markers.

#### Hierarchical cluster analysis

The phylogenetic tree was constructed using unweighted pair-group method with arithmetic mean (UPGMA) cluster analysis based on the p-distance model by MEGA 6.05 [30].

#### Bayesian model based cluster analysis

Bayesian clustering method implemented in STRUCTURE 2.3.3 [31] was used to infer population structure. These analyses were conducted based on an admixture model with correlated allele frequencies and no prior population origin information, with *K* varying from 1 to 10. Ten independent runs for each *K* were conducted using a burn-in length of 10,000 and a run length of 100,000 iterations. We determined the optimal *K* using ad hoc *ΔK* statistics described by Evanno et al. [32].

Individuals were assigned probabilistically to populations according to their membership coefficient (q). A graphical bar plot of membership coefficients was generated using DISTRUCT software [33].

#### Principal coordinate analysis

The genetic structure of the germplasm collection was analyzed by a principal coordinate analysis (PCoA) implemented by the GenAlEx 6.5 program. PCoA was based on standardized covariance of genetic distances calculated for markers.

#### Analysis of molecular variance and genetic differentiation

Analysis of molecular variance (AMOVA) was performed by decomposing the litchi accessions into four different fruit maturation-time groups using GenAlEx 6.5. The genetic differentiation between fruit maturation-time groups was analyzed using F_ST_ statistics by Arlequin 3.5 [34].

## Results

### Genetic diversity

Of the 148 genotyped SNP markers, 17 were monomorphic across the 96 litchi accessions, 19 generated SNP profiles with more than 10% missing data, and another 22 had MAF less than 5%. The remaining 90 SNP markers were used in the subsequent data analysis.

**Fig 1:**
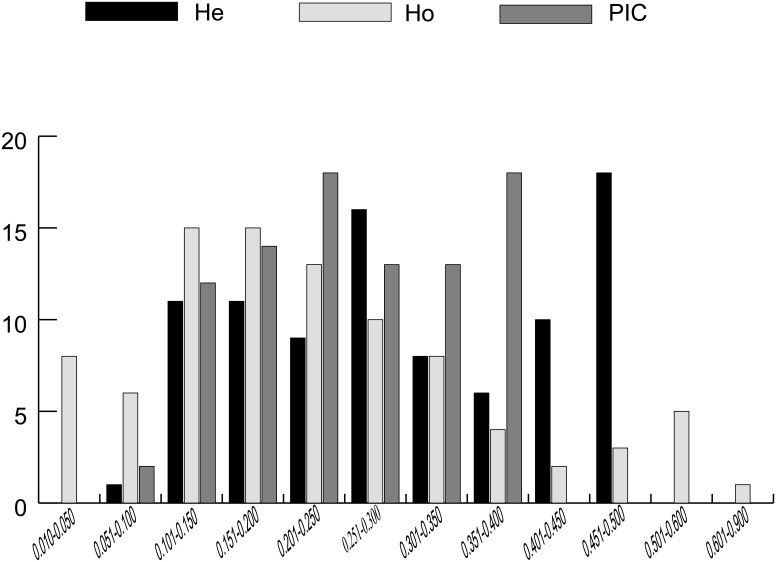
Distribution of expected heterozygosity (He), observed heterozygosity (Ho) and polymorphism information content (PIC).

The expected heterozygosity (He) and observed heterozygosity (Ho) ranged from 0.100 to 0.500 and 0.011 to 0.813, with an average of 0.305 and 0.232, respectively (Fig 1). In addition, the average PIC was 0.251, ranging from 0.094 to 0.375 (Fig 1).

### Statistical power of SNPs in litchi cultivar identification

Ninety-four different genotypes were identified among 96 litchi accessions based on the 90 SNPs. Two synonymous groups were identified, including ‘Heiye’ from Guangdong Province and ‘Wuye’ from Fujian Province, ‘Chengtuo’ from Guangdong Province and ‘Baitangli 1’ from Guangxi Province.

**Fig 2:**
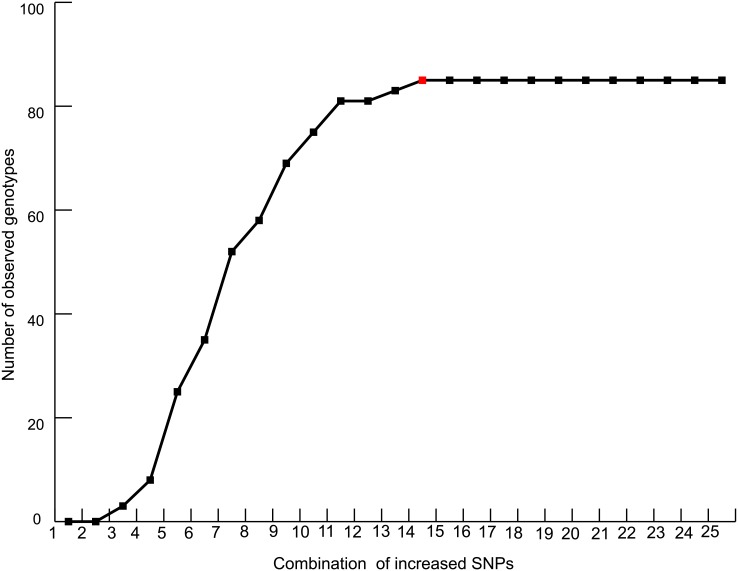
The relationship between observed genotypes and numbers of SNPs used.

Using 62 SNPs that had no missing data, 85 non-redundant genotypes were identified. The number of genotypes identified increased with the addition of SNPs, and 14 SNPs were enough to distinguish all 85 genotypes (Fig 2).

Stability of the 14 SNPs for cultivar identification was evaluated by the genotype analysis of 9 plants (on the average) for each of 10 cultivars. The 10 cultivars exhibited phenotypic diversity for several important litchi traits, including fruit color (green and red), presence of seeds (seeded and seedless), ripping period (early, medium, and late) [5]. The 10 cultivars also represented diverse geographical origins (Guangdong, Guangxi, Fujian, Hainan provinces). Furthermore, the 10 cultivars also displayed age divergence, including ancient cultivars that are more than a thousand years old (e.g., ‘Lingshanxiangli’), cultivars originating a few centuries ago (e.g., ‘Nuomici’), and those bred in the 21st century (e.g., ‘Nandaowuheli’).

Ninety-eight plants were analyzed with the selected 14 SNP set. Table 2 shows the genotypes obtained for each cultivar. Regarding the stability analysis, all the analyzed plants showed the expected genotype for the cultivars (Table 2). Therefore, the SNP marker set could be considered highly stable and a DNA barcode database could be established for the analyzed litchi accessions using the selected 14 SNP set.

**Table 2:** Genotypes for the 14 SNP set in the cultivars used for the stability study.

SNP name	SD	HY	NMC	QZHL	LSXL	YH	DCZ	LZ	XFZ	NDWHL
SNP3	A/G	G/G	G/G	G/G	G/G	A/G	A/G	A/G	G/G	G/G
SNP5	A/T	A/T	A/T	A/T	A/T	A/T	A/T	A/T	A/T	A/T
SNP39	A/T	T/T	T/T	T/T	A/T	A/T	A/T	T/T	T/T	T/T
SNP54	T/T	A/T	A/T	A/A	A/A	A/T	A/T	T/T	T/T	A/A
SNP57	T/T	C/T	C/C	C/T	C/C	C/T	C/T	C/T	C/C	C/C
SNP58	G/G	A/G	A/A	A/G	A/A	A/A	A/A	A/A	A/A	A/A
SNP60	T/T	T/T	T/T	T/T	A/A	T/T	T/T	T/T	T/T	T/T
SNP64	A/T	T/T	T/T	A/T	T/T	T/T	A/T	A/T	A/T	A/A
SNP83	A/G	A/G	A/A	A/G	A/A	A/A	A/A	A/G	A/G	A/G
SNP101	C/C	A/C	A/A	A/C	A/A	A/C	A/C	A/C	A/A	A/A
SNP103	C/C	T/T	C/C	C/C	C/C	C/C	C/C	C/T	C/C	C/C
SNP138	A/A	A/C	A/A	C/C	C/C	A/C	A/C	A/C	A/A	C/C
SNP146	A/A	A/G	A/G	A/G	A/G	A/G	A/G	A/G	G/G	A/G
SNP152	T/G	T/G	T/T	T/G	G/G	G/G	G/G	T/G	T/G	G/T

SD: Shuidong; HY: Heiye; NMC: Nuomici; QZHL: Qinzhouhongli; LSXL: Lingshanxiangli; YH: Yuanhong; DCZ: Dachenzi; LZ: Lanzhu; XFZ: Xiafanzhi; NDWHL: Nandaowuheli.

### Genetic relationships among accessions

**Fig 3:**
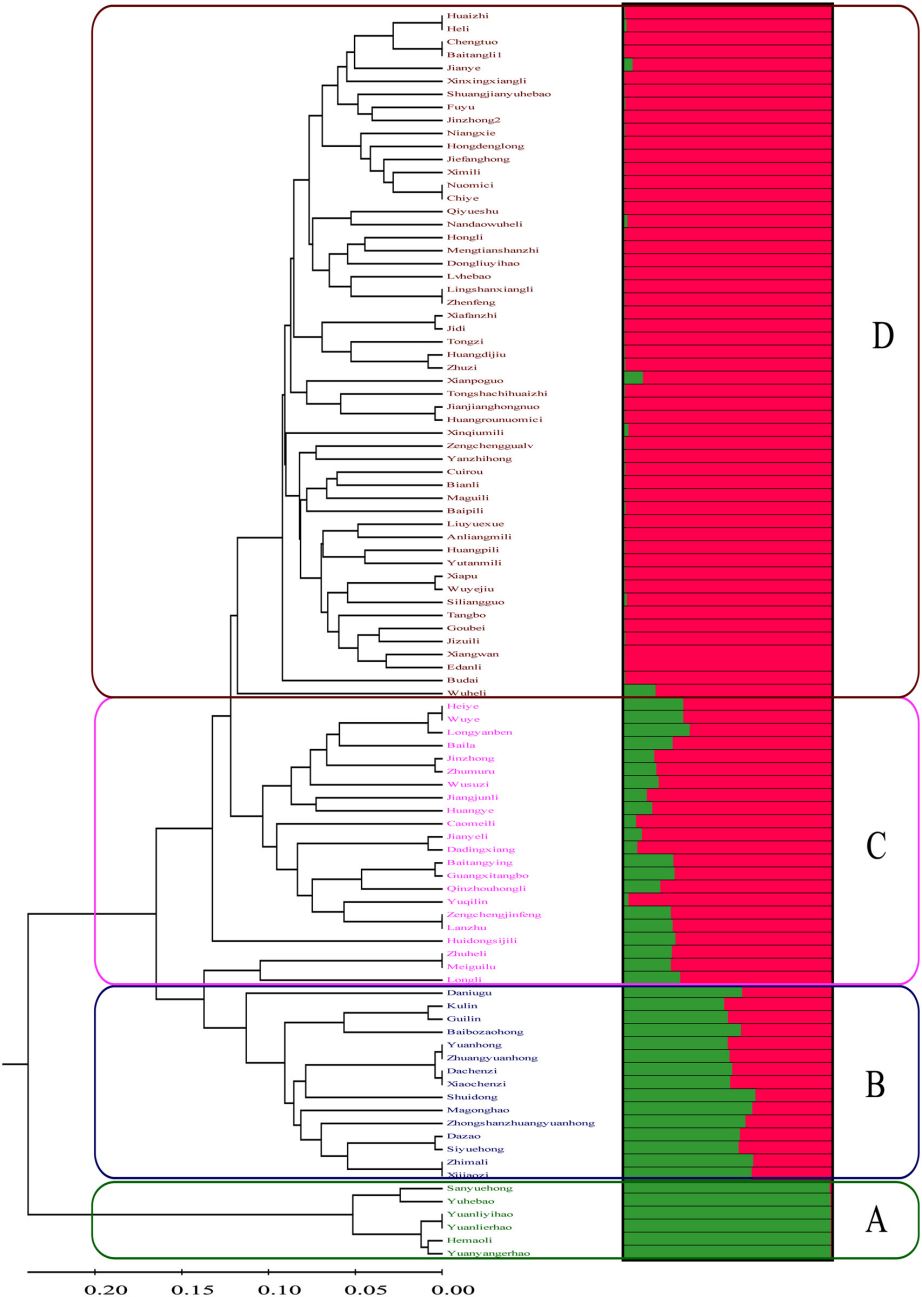
A phylogenetic tree and population structure of 96 litchi accessions based on 90 informative SNPs.

In order to analyze genetic relationships among accessions, a phylogenetic tree applying the UPGMA method was constructed based on the SNP genotype data of the 96 litchi accessions. The dendrogram revealed the classification of analyzed accessions into four clusters, and there was a perfect association between SNP genotypes and fruit maturation time since each cluster contained accessions of one maturation-time group (Fig 3). Cluster A included six accessions with extremely early-ripening time, that were ‘Sanyuehong’, ‘Yuhebao’, ‘Hemaoli’, ‘Yuanyangerhao’, ‘Yuanliyihao’, and ‘Yuanlierhao’; Cluster B contained 15 accessions with early-ripening time, such as ‘Dazao’, and ‘Shuidong’; Cluster C contained 22 accessions with middle-ripening time, such as ‘Heiye’, and ‘Baila’; Cluster D contained 53 accessions with late-ripening time, such as ‘Nuomici’, and ‘Huaizhi’ (Fig 3). Similar groups were also formed by neighbor-joining analysis (data not shown).

**Fig 4:**
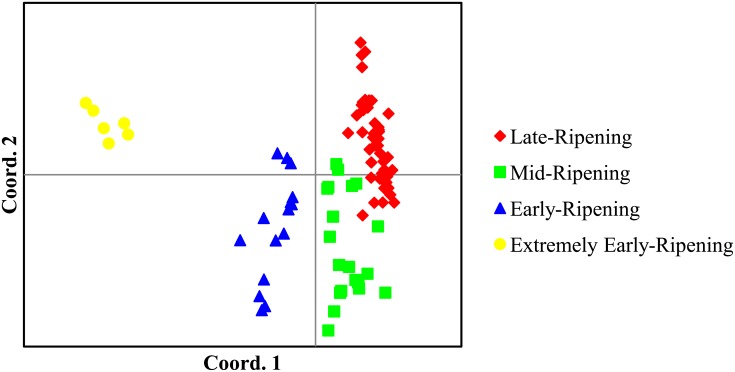
Two-dimensional PCoA plot based on 90 SNPs of litchi genotypes. Each dot represents one individual. The first two principal coordinates of PCoA account for 61.59% of the total variation.

In addition, principal coordinate analysis, which was performed as a complementary approach to display genotype clusters, also separated 96 litchi genotypes into four major clusters, which was consistent with assignments generated by the UPGMA clustering analysis (Fig 4). PCO 1 accounted for 50.81% of the genetic variance and separated accessions according to the fruit maturation time (early vs. late). PCO 2, accounting for 10.78% of the variation, further separated extremely-early ripening from early-ripening genotypes, and late-ripening from middle-ripening genotypes. Four germplasm groups were observed based on the first two PCO’s (Fig 4).

As a complement, the STRUCTURE analysis was used to reveal the number of subpopulations composing the genetic structure of the litchi germplasm collection. The maximum *ΔK* corresponded to *K* = 2, indicating that there were two major subpopulations. The membership coefficient (q) is presented in Fig 3 in comparison to the UPGMA dendrogram, which classified the collection into groups of closely related genetic accessions. Accessions with q values above 0.90 were assigned to a specific group, whereas accessions with q values lower than 0.90 were categorized as admixture forms with varying levels of membership shared between the two clusters. All six accessions with extremely early-ripening time were assigned to Cluster 1 with membership coefficients higher than 0.99, and most of the accessions with late-ripening time except samples ‘Wuheli’ (0.849), ‘Xianpoguo’ (0.91), ‘Jianye’ (0.96), and ‘Xinqiumili’ (0.98) were assigned to Cluster 2 with membership coefficients higher than 0.99 (Fig 3). All 15 accessions with early-ripening time were identified as admixture forms, which showed q values between 0.48 and 0.63 for Cluster 1. 18 of the 22 accessions with middle-ripening time were categorized as admixed, which showed q values between 0.68 and 0.89 for Cluster 2.

To confirm genetic structuring between studied litchi accessions, the partitioning of genetic variance between and within fruit maturation-time groups was assessed by AMOVA, which showed that 30% of the variation was between groups (P < 0.001) (Table 3). Genetic differentiation between fruit maturation-time groups was also tested using F_ST_ statistics estimated from pairwise comparisons. Pairwise comparisons showed considerable variation in the F_ST_ values, ranging from 0.153 to 0.625 (Table 4). The lowest genetic differentiation was between the late-ripening group and the middle-ripening group (F_ST_ = 0.153), and the highest genetic differentiation was between the late-ripening group and the extremely early-ripening group (F_ST_ = 0.625) (Table 4).

**Table 3:** Analysis of molecular variance (AMOVA) for 96 litchi accessions classified into four maturation-time groups.

Source of variation	df	Sum of squares	Percentage of variation	P value
Among groups	3	618.348	30%	0.000
Within groups	92	1122.735	6%	0.001
Within Individuals	96	997.000	64%	0.000

**Table 4:** Genetic differentiation (F_ST_) of litchi accessions according to fruit maturation time.

	Late-Ripening	Mid-Ripening	Early-Ripening	Extremely Early-Ripening
**Late-Ripening**	-			
**Mid-Ripening**	0.151	-		
**Early-Ripening**	0.294	0.164	-	
**Extremely Early-Ripening**	0.611	0.535	0.303	-

## Discussion

### SNP diversity in litchi

This is the first study in which the genetic diversity of 96 representative litchi genotypes in China was investigated using SNP markers. The genetic variation revealed among litchi accessions (He = 0.305) was higher than value for citrus based on SNPs [35], and comparable to the values obtained for grape (He = 0.304; [36]) and cacao (He = 0.367; [37]) also using SNP markers, suggesting abundant diversity in Chinese litchi germplasm. This diversity could be attributed to the reproduction mode (open pollination) and breeding method (selection of landraces) of litchi cultivars in China. The high level of genetic diversity indicates rich germplasm variation, allowing for the selection of more beneficial genes to boost genetic improvement.

In general, diversity values (He) for SNPs are lower than those estimated for SSR markers because of the SNP marker’s bi-allelic nature [38–41]. However, the diversity revealed among litchi accessions in the present study (He = 0.305) was higher than that previously reported in a study using SSR markers (He = 0.27, [18]), which also analyzed 96 litchi accessions in China, with 49 of the same accessions as the present study. The different number of markers used in these two studies might explain these results, as there were 90 polymorphic SNPs in our study, whereas only 30 SSRs were employed by Xiang et al. [18]. Therefore, our results demonstrate that the drawback of SNP markers in terms of their relatively lower level of diversity can be overcome by using larger sets of markers.

### SNP markers for litchi cultivar identification

As the largest litchi-producing country, the use of homonyms and synonyms for cultivar designation is a big problem in Chinese litchi genotypes. Several previous studies have reported many synonyms and homonyms in litchi collections in China [9–11, 17], making it difficult to identify the reference of litchi cultivars. Our results identified two groups of cultivars that were identical in all 90 SNP loci tested. One of the groups was formed by ‘Heiye’ from Guangdong Province, and ‘Wuye’ from Fujian Province. This was in agreement with expectations, as ‘Wuye’ is an alias of ‘Heiye’, and ‘Wuye’ was introduced to Fujian Province from Guangdong Province about 500 years ago according to ancient Chinese literature [5]. Actually, these two cultivars have almost the same morphological and biological characteristics [5] and have been suspected as same cultivar for many years. Therefore, our results confirmed that these two cultivars are synonyms. Another group was formed by ‘Chengtuo’ from Guangdong Province, and ‘Baitangli 1’ from Guangxi Province. This was unexpected based on the origin of both cultivars; however, they share great similarity in tree and fruit characteristics as well as ripping period [5], indicating that they could be synonymous cultivars or sports (spontaneous somatic mutants).

Due to the problem of homonyms and synonyms in litchi cultivar nomenclature, accurate characterization of litchi germplasm is a primary concern. Molecular markers are the chosen methods for cultivar identification in many crops [42–46]. As for litchi germplasm in China, Yi et al. [12] constructed a DNA fingerprint database for 39 litchi cultivars using three AFLP primers, and Fu [17] established SSR fingerprints for 36 litchi germplasm resources using two primer pairs. However, the number of litchi cultivars used in those studies was limited, and the DNA fingerprints were based on the results of electrophoresis, which is an important drawback both for cultivar identification in routine practices and data comparison among different laboratories.

In this study, we investigated the possibility of using SNP markers for litchi cultivar identification. Our results showed 14 SNPs were sufficient to distinguish the 85 non-redundant litchi genotypes. These SNPs were found to be highly stable by repeated analyses in a selected group of cultivars. Therefore, the SNP genotypes in these 14 SNPs could be used as reference genetic barcodes for each analyzed litchi accession. As SNPs are bi-allelic, allele identification and genotype recording are always comparable among different genotyping platforms and laboratories, the litchi SNP barcode database constructed in this study allowed for the standardization of cultivar nomenclature.

In the course of litchi’s dissemination to other countries, there has been considerable confusion regarding the identity of the original Chinese litchi cultivars, owing to misidentification or mislabeling, the corruption in the English transliteration of Chinese dialects, the replacement of the original Chinese names by local names, and the poor documentation of passport information during germplasm introduction [47–53]. Only comparisons with Chinese cultivars from their home ranges would help to ensure the correct identity of litchi collections in other countries. As a result, the litchi SNP barcode database constructed in this study would greatly facilitate the integration and interpretation of litchi germplasm across different genebanks in litchi-producing countries, to have a comprehensive picture of current litchi germplasm conservation worldwide.

### Genetic relationships and population structures among litchi accessions

Understanding the genetic relationships and population structures of litchi germplasm is crucial for litchi breeding projects by facilitating the selection of optimal parental combinations, and for germplasm management to avoid genetic redundancy. Traditionally, the most commonly used classification criterion for Chinese litchi accessions is the shape of skin segments and protuberances, based on which the germplasm are divided into three types: smooth protuberances (e.g., ‘Heiye’), protruding and hard protuberances (e.g., ‘Nuomici’), and hair-like protuberances (e.g., ‘Dazao’) [5]. However, the phylogenetic tree constructed in this study revealed no clear relationship between the shape of skin segments and protuberances and the genomic composition, indicating this classification system failed to reflect the genetic relationships among litchi germplasm.

In contrast, the UPGMA dendrogram revealed four groups of litchi genotypes that were congruent with fruit-maturation time, and similar results were reported in Liu and Mei [10], and Fu [17]. Based on RAPD technology, Liu and Mei [10] found that 60 Chinese litchi accessions could be separated into three groups, which corresponded to extremely early maturation, early-to-middle maturation, and late to extremely late maturation traits. Using SSR markers, Fu [17] also found that 47 Chinese litchi accessions showed evidence of clustering according to fruit-maturation periods. The dendrogram in this study was established using 90 SNPs evenly spaced across the litchi genome for 96 litchi accessions collected from all over China, which was based on a broader genomic infrastructure and more representative germplasm collections. Together with the results from the present study, it can be proven that fruit-maturation period is a better indicator for classifying phylogenetic relationships among litchi accessions than the shape of skin segments and protuberances. Therefore, fruit-maturation period should be considered as the primary criterion for litchi taxonomy.

In our UPGMA dendrogram, six accessions with extremely early-ripening time grouped together remotely from the other litchi accessions, indicating a divergent origin. Similar results were reported in Liu and Mei [10] who found extremely early litchi accessions formed a narrow group that was distant from other litchi groups. In addition, Degani et al. [50] also found that ‘Third Month Red’, which is the translated name of the extremely early-maturing ‘Sanyuehong’ in the present study, was the most divergent accession among the 66 litchi accessions in germplasm collections in Israel. Therefore, considering its biological and genetic distinctiveness, our results supported the proposal that classified litchi accessions with extremely early-ripening time into a varietas under *Litchi chinensis* Sonn. [10].

The model-based STRUCTURE analysis revealed the presence of two clusters among the collected litchi genotypes. Accessions with extremely early-ripening and late-ripening time were assigned to Cluster 1 and 2 with nearly total ancestry, respectively. However, accessions with early-ripening and middle-ripening times were identified as admixed individuals with varying levels of membership shared between these two clusters, indicating a hybrid origin of these accessions during litchi domestication. The evolution of cultivated litchi regarding its geographic origin and number of domestication has been historically unclear [54]. Therefore, our future research will focus on the genetic analysis of all wild litchi populations within their natural distribution ranges, to further understand the origin of litchi.

## Supporting Information

S1 Table Plant samples used for the stability studies of the 14 SNP set. (XLSX)

S2 Table SNP name, linkage group and SNP alleles of 155 SNPs used in this study. (XLSX)
